# Fetal outcomes and their correlates following caesarian section in a rural setting in Ghana

**DOI:** 10.1371/journal.pone.0293029

**Published:** 2023-10-31

**Authors:** Eugene Sackeya, Martin Muonibe Beru, Richard Nomo Angmortey, Douglas Aninng Opoku, Victoria Achiaa Boamah, Francis Appiah, Aliyu Mohammed

**Affiliations:** 1 Department of Epidemiology and Biostatistics, School of Public Health, Kwame Nkrumah University of Science and Technology, Kumasi, Ghana; 2 Tatale District Hospital, Tatale, Ghana; 3 Tamale Teaching Hospital, Tamale, Ghana; 4 Department of Global Health and International Health, School of Public Health, Kwame Nkrumah University of Science and Technology, Kumasi, Ghana; 5 Allen Clinic, Family Healthcare Services, Kumasi, Ghana; 6 School of Health and Social Care, Swansea University, Swansea, United Kingdom; 7 Department of Social Sciences, Berekum College of Education, Berekum, Bono Region, Ghana; Bangabandhu Sheikh Mujib Medical University (BSMMU), BANGLADESH

## Abstract

**Background:**

Regular evaluation of caesarean section (CS) is required due to their rising trend and outcomes. Many women recently opt for elective CS, even in resource-constrained settings. Data evaluating the outcomes of CS is however sparse. Hence, this study sought to determine the rate of fetal mortalities and their determinants following CS in the Tatale District Hospital of the Northern Region, Ghana.

**Methods:**

A retrospective cross-sectional study was employed to analyze the medical records of 275 women who underwent CS from 2019 to 2021. Data were collected from the hospital’s record of CS cases from 2019 to 2021. Descriptive statistics were used to summarize the data and Pearson’s chi-square/Fisher’s exact test was used to examine the relationship between maternal and obstetric characteristics and fetal mortality. At a 95% confidence interval (95% CI), logistic regression was fitted to assess significant variables and reported the results using odds ratio.

**Results:**

Of 1667 deliveries, 16.5% of the mothers gave birth by CS. A fetal mortality rate of 76.4 per 1000 total births was recorded following CS. Babies born with low Appearance, Pulse, Grimace, Activity and Respiration (APGAR) scores (0–3) at fifth-minute had an increased risk of fetal mortality (AOR  =  523.19, 95%CI: 49.24–5559.37, p  =  <0.001). Having a history of previous CS, cephalopelvic disproportion and delayed labour were the major indications for CS.

**Conclusion:**

Overall, this study found a high rate of CS based on the World Health Organization‘s recommended CS rate. Interventions such as reducing the waiting time for surgery and early diagnosis of the need for CS, and ensuring the availability of modern equipment to resuscitate infants with low APGAR scores can significantly improve fetal outcomes following CS.

## Introduction

A life-saving surgical operation known as a caesarean section (CS) is performed when specific difficulties during pregnancy and labour occur [1]. CS has increased worldwide in recent decades, especially in middle- and high-income countries [2] with rate ascendancy from an estimated 7% in 1990 to 21% in 2021, and is expected to rise further in the coming decade[3]. Inequalities in the use of CS, both between and within nations, as well as the expenditures that unneeded CS imposes on financially strapped health systems, are issues up for debate [4]. Worldwide, CS rates in Eastern Asia (63%), Latin America and the Caribbean (54%), Western Asia (50%), Northern Africa (48%), Southern Europe (47%), and Australia and New Zealand (45%) have been predicted to have the highest rates by 2030 [5]. In resource-constrained areas such as sub-Saharan Africa, having access to safe CS is a key strategy for lowering maternal and neonatal mortalities [6]. Africa has the lowest CS rate in the world with only 7.3% of newborns delivered through this method [7]. Egypt (51.8%) and Mauritius (47%) have the highest CS rates in Africa [7].

Caesarean section can effectively prevent maternal and fetal mortality and morbidity when medically necessary [3]. However, there is little proof that caesarean birth is beneficial to women or infants who do not require it [5]. A review of national data from 159 countries indicated that countries with national CS delivery rates of 5% to 10% had the lowest neonatal and maternal mortality rates [1, 8]. However, the benefits of CS delivery reduces when the rates exceed 10% [8]. This indicates that high CS delivery rates might not have additional benefits in terms of reducing mortality. In all regions, the WHO recommends that the CS delivery rate not exceed 15% of all deliveries [3].

A survey in Ghana reported that nearly 18.5% of women who delivered in health facilities 5 years preceding the survey delivered their babies through CS [9]. While it is not expected that CS rates will be the same across all facilities, regional variations in CS rates have been observed in Ghana, with the Greater Accra Region recording the highest rate, with nearly one in four women (23%) undergoing CS. The following rates have also been observed in the Volta (15%), Ashanti (12%), Eastern (12%), Brong-Ahafo (12%), and Central (11%) Regions in Ghana [9]. Between 1998 and 2014, Ghana had differences in the number of CS births, which grew over time [10]. A study also showed that fetal weight of 4kg and above along with referred pregnant women are the leading indications for CS in northern Ghana [11]. The majority of Ghanaian women (87%) had spontaneous vaginal deliveries whiles about 55% of those who had CS for their most recent birth had an elective rather than an emergency procedure [12]. In a study of women giving birth at the University of Cape Coast Teaching Hospital in Ghana, it was discovered that babies born via emergency CS had a considerably greater rate of adverse fetal outcomes [13].

The present study was conducted to determine the prevalence and factors associated with fetal mortality following CS. This study also sought to estimate the prevalence of CS at the Tatale District Hospital. Considering the complex situations of unmet needs and overuse of CS, coupled with unsafe surgical practices and the non-availability of essential medical equipment for CS operations [1], this study provides significant data for designing public health interventions targeted at improving the outcomes of CS births as well as improving maternal and child health in Ghana and beyond.

## Methods

### Study design and setting

This was a retrospective analytical cross-sectional study that included 275 women who delivered through CS at the Tatale District Hospital, Ghana. The facility provides medical service to the residents of Tatale Sanguli District, parts of the Republic of Togo, and other neighbouring districts in the Northern Region of Ghana. It also serves as the first point of call for all emergency cases from all sub-district health facilities. Due to the inadequate number of trained midwives in the district, community health nurses augment the efforts of the midwives. The hospital has consistently had only one medical officer who attends to surgical cases such as CS.

The Tatale District Hospital is limited in terms of advanced medical equipment. The facility lacks ultrasonographic machines for diagnosing and assessing pregnancy states in addition to the non-availability of a Neonatal Intensive Care Unit (NICU), a monitoring machine for anaesthesia care and resuscitation devices and an inadequate supply of oxygen. This can affect the quality of CS conducted in the facility.

### Variables description

#### Dependent variable

The key outcome variable was fetal mortality. This was defined as the fetus delivered dead following the CS or died shortly after the operation that is before the mother was moved from the theater to the recovery room. The secondary outcome was the rate of CS. This was defined as the proportion of women who delivered through CS divided by the total number of women who delivered in the hospital for the period expressed as a percentage.

#### Independent variables

Ten (10) explanatory variables were selected for the study. These were Appearance, Pulse, Grimace, Activity and Respiration (APGAR) score at the fifth minute, gravidity, parity and gestational age. APGAR score was a composite index obtained from the five cardinal measures at the fifth minute after delivery. An APGAR score at the fifth minute of 4 and above were considered to be moderate to high score whiles a fifth-minute score of 0–3 was considered low [14]. Gravidity indicates the number of times a participant has been pregnant prior to the current pregnancy whilst parity explains the number of times a participant has given birth before the current delivery. The gestational age describes the number of weeks a participant carried a pregnancy before delivery. The mother’s haemoglobin (HB) level indicates the HB level of the mother as recorded in the theatre records book, specifically the HB taken during the process of preparing the woman for the CS. Baby weight refers to the initial weight recorded at birth whiles the time between admission and CS refers to the period between the arrival of participants at the health facility and when the CS was done. Also, antenatal care (ANC) visits indicate the number of times a mother attended ANC before delivery. Type of anaesthesia was defined as whether a pregnant woman was given general anaesthesia or spinal anaesthesia prior to CS while type of CS indicates whether the surgery done was a planned one (elective) or emergency. These variables were selected due to their theoretical and practical significance to neonatal/fetal health [15, 16].

#### Data sampling, collection, and management

The census technique was used to review medical records of all CS cases documented in the theatre register from 1^st^ January 2019 to 31^st^ December 2021. This period was purposively selected to assess the fetal mortalities following CS of all cases. The reason for selecting this period was the constant availability of medical officers qualified to provide CS service to pregnant women who may need it. The key variables of interest extracted for the study included maternal age, parity, gravida, educational level, haemoglobin level and employment status. The type of CS delivery (emergency or elective CS), type of anaesthesia (general or spinal), the fetal weight at birth and the time between admission and the CS were also retrieved from the theatre register. The data were retrieved using a predesigned checklist (designed using Microsoft Excel software). Only variables with missing values less than ten per cent were considered with missing values manually imputed using either the mean, median or the mode.

Data retrieved from the theatre register were compared with the labour room register and the ANC register to ensure accuracy, consistency and also to track and fill all missing data from the records in the theatre. The data was saved on a computer hard drive and also in a Google drive for use and protection.

### Statistical analysis

STATA statistical software version 16.0 was used to analyze the data. Descriptive statistics using mean, standard deviation, frequencies, and percentages were used to describe categorical and continuous variables. Cross-tabulation computation was done for the outcome variable across the key explanatory variables. At a cut-off p-value of ≤0.05, a chi-square test of independence or Fisher exact test was calculated between the outcome variable and key explanatory variables and those that were not significant were not entered into the multivariate analysis. At a 95% confidence interval (95% CI), two logistic regression models were fitted between the outcome variable and key explanatory variables. The first model (Model I) explored a bivariate association whilst Model II followed a multivariate approach. The results were reported in crude odds ratio (COR) and adjusted odds ratio (AOR) for Model I and Model II respectively. A multi-collinearity test was performed for the key explanatory variables using the Variance Inflation Factor (VIF). The VIF results indicated no evidence of multi-collinearity between the explanatory variables (Mean VIF = 1.06, Maximum VIF = 1.12 Minimum VIF = 1.02) (see S1 Table). Finally, the Hosmer-Lemeshow test was applied to measure the model fit which indicated no evidence of poor fit (P = 0.72).

## Results

A total of 1667 deliveries were recorded at the hospital within the period. The mean age of the mothers was 27.9 (±**7**.2) years. Out of the 1667 deliveries recorded, 275 (16.50%) were born by CS out of which 21 (7.64%) fetal mortalities were recorded (fetal mortality rate of 76.4 per 1000 total births). Majority (89.1%) of children delivered had APGAR score recordings from 4 to 10 in the fifth minute and about one-tenth (10.9%) recorded 0 to 3 APGAR scores in the fifth minute after delivery. About one-fifth (19.3%) had their CS as a result of cephalopelvic disproportion (CPD) [Table 1].

**Table 1:** Study participants’ characteristics and Fetal outcome (N = 275).

	Univariate		Bivariate		
Variable		Fetal outcome			
	n (%)	Alive n (%)	Died n (%)	X^2^	(p-value)
**Age Group**					**0.876** ^*^
15–19	28 (10.2)	27 (96.4)	1 (3.6)		
20–34	187 (68.0)	172 (92.0)	15 (8.0)		
35–49	83 (21.8)	55 (91.7)	5 (8.3)		
Mean age (±SD)	**27.9 (**±**7.2)**				
**NHIS Status**					**0.382** ^*^
Insured with NHIS	269 (97.8)	249 (92.6)	20 (7.4)		
Not insured with NHIS	6 (2.2)	5 (83.3)	1 (16.7)		
**Employment status**				**0.12**	**0.726**
Employed	187 (68)	172 (92.0)	15 (8.0)		
Unemployed	88 (32)	82 (93.2)	6 (6.8)		
**Occupation (n = 187)**					**0.781** ^*^
Civil servants	4 (2.1)	4 (100)	0 (0.0)		
Farmers	110 (58.8)	99 (90.0)	11 (10.0)		
Traders	41 (21.9)	39 (95.1)	2 (4.9)		
**Level of Education**					**0.941** ^*^
No formal education	185 (67.3)	169 (91.4)	16 (8.7)		
Primary level	14 (5.1)	14 (100.0)	0 (0.0)		
JHS level	29 (10.6)	27 (93.1)	2 (6.9)		
SHS level	38 (13.8)	35 (92.1)	3 (7.9)		
Tertiary level	9 (3.3)	9 (100.0)	0 (0.0)		
**Gravidity**				**0.14**	**0.708**
Primigravida	74 (27.3)	69 (93.2)	5 (6.8)		
Multigravida	197 (72.7)	181 (91.9)	16 (8.1)		
**Parity**					**0.798** ^*^
Primiparous	131 (48.3)	122 (93.1)	9 (6.9)		
Multiparous	84 (31.0)	76 (90.9)	8 (7.1)		
Grand multiparous	56 (20.7)	52 (92.9)	4 (7.1)		
**Type of CS**					**0.618** ^*^
Emergency CS	269 (97.8)	248 (92.2)	21 (7.8)		
Elective CS	6 (2.2)	6 (100.0)	0 (0)		
**Type of anaesthesia**					**0.085** ^*^
Spinal anaesthesia	252 (91.6)	235 (93.3)	17 (6.7)		
General anaesthesia	23 (8.4)	19 (82.6)	4 (17.4)		
**Baby APGAR score at 5**^**th**^ **minute**					**<0.001** ^*^
Low (0–3)	30 (10.9)	10 (33.3)	20 (66.7)		
Moderate/high (4–10)	245 (89.1)	244 (99.6)	1 (0.4)		
**ANC visits**				**7.27**	**0.007**
Less than 4 visits	38 (13.8)	31 (81.6)	7 (18.4)		
4 or more visits	237 (86.2)	223 (94.1)	14 (5.9)		
**Gestational age**				**4.75**	**0.029**
Pre-term	75 (27.3)	65 (86.7)	10 (13.3)		
Term/ Post-term	200 (72.7)	189 (94.5)	11 (5.5)		
**Mother’s HB level**					**0.031** ^*^
Below 7.6	17 (6.2)	13 (76.5)	4 (23.5)		
7.6 and above	258 (93.8)	241 (93.4)	17 (6.6)		
**Time b/n admission and CS**					**0.002** ^*^
Within 30 minutes	11 (4.0)	9 (81.8)	2 (18.2)		
Within 2 hours	69 (25.1)	58 (84.1)	11 (15.9)		
More than 2 hours	195 (70.9)	187 (95.9)	8 (4.1)		
**Baby’s weight at birth**				**6.65**	**0.031**
Low weight	55 (20.0)	47 (85.5)	8 (14.5)		
Normal weight	220 (80.0)	207 (94.1)	13 (5.9)		

SD = standard deviation, NHIS = national health insurance scheme, JHS = junior high school, SHS = senior high school, CS = caesarean section, ANC = antenatal care, b/n = between

* = Analyzed using Fisher’s exact test

From the bivariate analyses, the baby’s APGAR score at the fifth minute was found to be associated with fetal mortality. Other variables that were found to be associated with fetal mortality included the number of ANC visits by mothers, time between admission and CS as well as the baby’s birth weight [Table 1].

### Indicators for CS

Of the total number of CS, 19.3% were as a result of cephalopelvic disproportion. Also, 17.5% were due to the mother’s history of previous CS and delayed labour accounted for 15.3% of CS operations. Almost 1 in every 10 fetal mortalities (8.27%) occurred among mothers who presented with antepartum haemorrhage. Meanwhile, postdated pregnancies, mothers with bad obstetric history and multiparity recorded no fetal mortalities [Table 2].

**Table 2:** Indications for caesarean section.

Indication for CS	Total n (%)	Died n (%)	Alive n (%)
Antepartum haemorrhage	23 (8.4)	9 (3.3)	14 (5.1)
Bad obstetric history	7 (2.6)	0 (0.0)	7 (2.6)
Cephalopelvic disproportion	53 (19.3)	3 (1.1)	50 (18.2)
Delayed labour	42 (15.3)	2 (0.7)	40 (14.6)
Distress mother/child	25 (9.1)	2 (0.7)	23 (8.4)
Malpresentation	18 (6.6)	1 (0.4)	17 (6.2)
Medical condition	11 (4.0)	1 (0.4)	10 (3.6)
Multiparous	7 (2.6)	0 (0.0)	7 (2.6)
Post date	22 (8.0)	0 (0.0)	22 (8.0)
Twin gestation	8 (2.9)	1 (0.4)	7 (2.6)
Previous CS	48 (17.5)	2 (0.7)	46 (16.7)
Others	11 (4.0)	0 (0.0)	11 (4.0)

### Predictors of fetal mortalities

Children who were born preterm, mothers who visited the ANC less than 4 times, participants with HB level of 7.5g/dl, and women who were admitted within 30 minutes before the CS due to delays in reporting to the hospital in time were found to be statistically associated with fetal mortalities at the bivariate level (Model I). After adjusting for other covariates (Model II), children born with fifth minute APGAR score (0–3) had higher odds of fetal mortality compared to those with fifth minute APGAR score of 4–10 (AOR  =  523.19, 95%CI: 49.24–5559.37, p  =  <0.001) [Table 3].

**Table 3:** Logistic regression results of predictors of fetal mortalities.

	Model I		Model II	
Variables	COR (95%CI)	P-Value	AOR (95%CI)	P-Value
**Baby APGAR score**				
Moderate/high (4–10)	Ref			
Low (0–3)	487.99 (59.43–4007.32)	<0.001)	523.19(49.24–5559.37)	<0.001
**ANC visits**				
4 or more visits	Ref			
Less than 4 visits	3.60 (1.35–9.60)	0.011	2.68 (0.39–18.65)	0.319
**Gestational age**				
Term/Post-term	Ref			
Pre-term	2.64 (1.07–6.51)	0.035	1.61 (0.29–8.95)	0.586
**Mother’s HB(g/dl)**				
7.6 and above	Ref			
7.5 and below	4.36 (1.28–14.83)	0.018)	6.28 (0.30–8.94)	0.236
**Time b/n admission and CS**				
More than 2 hours	Ref			
Within 2 hours	4.43 (1.70–11.55)	0.002	0.95 (0.19–4.86)	0.951
Within 30 minutes	5.19 (0.96–28.07)	(0.056)	0.88 (0.05–15.90)	0.929
**Baby weight at birth(kg)**				
**Normal weight**	Ref			
Low weight	2.71 (1.06–6.91)	0.037	0.89 (0.14–5.56)	0.901
Number of observations		**275**		
Model Fit Testing (Hosmer-Lemeshow)		**2.87(0.72)**		

95% confidence intervals in brackets, COR = Crude Odd Ratio, AOR = Adjusted Odds Ratio, Ref = Reference category

## Discussion

The remarkable and consistent growth in the prevalence of CS over the past few decades has prompted more inquiry, discussion, and worry among healthcare professionals, governments, legislators, scientists, and clinicians [1, 17].

The rate of CS (16.5%) in the present study is high compared with the 10% to 15.0% recommended by the WHO [1] but similar to the national rate (16.0%) that was reported in the Ghana Demographic Health Survey and an earlier study (16.0%) in 2017 [18]. However, the CS rate observed in the present study is lower than the 26.9% reported in a study in the Cape Coast, Ghana [13]. The high rates of CS above WHO recommendation in this study may be due to the hospital serving as a referral centre for clinics, health centres, and Community-based Health Planning and Services (CHPS) compounds, as well as referrals from neighbouring districts.

There were 76.4 fetal mortalities per 1000 total births following CS within the study period. This rate was found to be relatively higher compared to a study conducted in Ethiopia, which found 55.6 fetal mortalities per 1000 total births following CS [19]. The difference in the mortality rate could be attributed to the differences available in the two facilities including human resources, infrastructure and other health dynamics. The fetal mortality rate in the Tatale District Hospital following CS was also higher when compared with rates among women with combined delivery methods in a study which sort to compare if facility birth reduced maternal and fetal mortality in Brong Ahafo, Ghana [20]. The reason for this variation could be that most of the facilities within the Brong Ahafo have more skilled staff and modern equipment compared to the Tatale District Hospital.

The most common indicators for CS in the district hospital included cephalopelvic disproportion, previous CS, delayed labour and distressed mother/baby. These findings are relatively similar to an earlier study in Ghana which had previous CS, big baby, failure in progress and fetal distress being the most common indicators for CS [13]. Similar to a study conducted in a teaching hospital in Burkina Faso, our study found that women who suffered antepartum haemorrhage recorded the highest fetal mortalities making it the most dangerous indicator requiring early diagnosis and intervention [21]; hence, the need to train midwives, nurses and other practitioners on the early sign of antepartum haemorrhage and its management.

The study revealed that children born with low APGAR scores (0–3) at the 5^th^ minute had higher odds of fetal mortality compared with children with 4 and above APGAR scores in the 5^th^ minute. This finding is similar to a study that sort to find the association between APGAR scores of 7 to 9 and neonatal mortality and morbidity in a population-based cohort study of term infants in Sweden [22]. The APGAR score is an expression of the infant’s physiologic condition at one point in time, which includes subjective components. Numerous factors can influence the Apgar score, including maternal sedation or anaesthesia, congenital malformations, gestational age, trauma, and interobserver variability.[23]. Paying attention to these factors and taking the necessary steps to avoid them can significantly improve APGAR scores and increase infant survival rates.

### Strengths and limitations of the study

The findings in the study are interpreted in the light of the following. Because this is a retrospective study, several critical obstetric outcomes and indications may not have been consistently reported, preventing further subanalysis. As an index study to determine the fetal outcomes following CS at the facility level, the findings give considerable evidence for the current situation and serve as the foundation for future research.

This study was conducted in one setting and thus findings cannot be generalized to represent other health facilities in Ghana, and another notable is the relatively small sample size, which reduces the statistical power and the presence of missing data. One other limitation is the large confidence intervals. This means the results with large confidence intervals should be interpreted with caution as it reduces precision.

## Conclusion

The present study recorded high rates of CS (16.5%) and fetal mortality of 76.4 per 1000 total births following CS. The study found a low APGAR score (0–3) at the fifth minute to be significantly associated with fetal mortality. We recommend a refresher training on early signs and effective management of labour for all midwives and other auxiliary staff who augment the labour workforce. We also recommend a multi-sector collaboration to address some of the challenges identified i.e., ANC attendance, low birth weight, early reporting to the hospital and issues relating to maternal haemoglobin levels.

## Supporting information

S1 Table Multi-collinearity test results. (DOCX)

S1 Dataset (XLSX)
