# Supplementary material for: Fetal outcomes and their correlates following caesarian section in a rural setting in Ghana
Source: PLoS One. 2023 Oct 31;18(10):e0293029. doi: 10.1371/journal.pone.0293029 (PMC10617686; doi:10.1371/journal.pone.0293029)
Supplement: S1 Table — (DOCX) [file pone.0293029.s001.docx]

**S1 Table:** **Multi-collinearity test results**

Variable VIF 1/VIF R-Squared

Apgar score 1.12 0.8913 0.1087

Gestational age 1.06 0.9461 0.0539

ANC visit 1.03 0.9678 0.0322

Baby weight at birth 1.06 0.9418 0.0582

Mother’s HB level 1.02 0.9786 0.0214

Time b/n admission and CS 1.08 0.9270 0.0730

**Mean VIF 1.06**
