# Supplementary material for: Fetal outcomes and their correlates following caesarian section in a rural setting in Ghana
Source: PLoS One. 2023 Oct 31;18(10):e0293029. doi: 10.1371/journal.pone.0293029 (PMC10617686; doi:10.1371/journal.pone.0293029)
Supplement: Attachment — Submitted filename: PONE-D-23-05404_reviewercomments.pdf [file pone.0293029.s003.pdf]

# PLOS ONE

# Foetal outcomes and their correlates following caesarian section in a rural setting in Ghana

--Manuscript Draft--

| | |
|---|---|
| **Manuscript Number:** | PONE-D-23-05404 |
| **Article Type:** | Research Article |
| **Full Title:** | Foetal outcomes and their correlates following caesarian section in a rural setting in Ghana |
| **Short Title:** | Foetal outcomes following caesarian section delivery in Ghana |
| **Corresponding Author:** | Eugene Sackeya, MPhil<br>Tatale District Hospital<br>Tatale, Northern Region GHANA |
| **Keywords:** | Foetal outcomes; correlates; Caesarian section; Ghana; mortality |
| **Abstract:** | Background: Regular evaluation of caesarean section (CS) is required due to the rising trend in CS and their outcomes. Many women recently opt for elective CS, even in resource-constraint settings. Data evaluating the outcomes of CS is however sparse. Hence, this study sought to determine the rate of perinatal foetal outcomes and their determinants following CS in the Tatale District Hospital of the Northern Region, Ghana.<br>Methods: A retrospective cross-sectional study was employed to analyze medical records of 275 women who underwent CS from 2019 to 2021. Data was collected from the hospital's record on CS cases from 2019 to 2021. Descriptive statistics were used to summarise the data and Pearson's chi-square/Fisher's exact test was used to examine the relationship between maternal and obstetric characteristics and foetal outcomes. At 95% confidence interval (95% CI), logistic regression was fitted to assess significant variables and reported the results using odds ratio.<br>Results: Of 1667 deliveries, 16.5% of the mothers gave birth by CS. Of this, 7.64% of babies were stillbirths. Babies born with poor Appearance, Pulse, Grimace, Activity and Respiration (APGAR) scores had an increased risk of infant mortality (AOR:523.19 95% CI: 49.24-555.37). Having a history of previous CS, cephalopelvic disproportion and delayed labour were the major indictors for CS.<br>Conclusion: Overall, this study found a high rate of CS based on World Health Organization 's recommended CS rate. Interventions such as reducing the waiting time for surgery and early diagnosis of the need for CS, and ensuring the availability of modern equipment to resuscitate infants with low APGAR scores can significantly improve foetal outcomes following CS. |
| **Order of Authors:** | Eugene Sackeya |
| | Martin Muonibe Beru |
| | Richard Nomo Angmortey |
| | Douglas Aninng Opoku, MPH |
| | Victoria Achiaa Boamah |
| | Francis Appiah |
| | Aliyu Mohammed, PhD |
| **Additional Information:** | |
| **Question** | **Response** |
| **Financial Disclosure**<br><br>Enter a financial disclosure statement that describes the sources of funding for the work included in this submission. Review | The authors received no specific funding for this work |

*Powered by Editorial Manager® and ProduXion Manager® from Aries Systems Corporation*

| | |
|---|---|
| the submission guidelines for detailed requirements. View published research articles from *PLOS ONE* for specific examples.<br><br>This statement is required for submission and **will appear in the published article** if the submission is accepted. Please make sure it is accurate.<br><br>**Unfunded studies**<br>Enter: *The author(s) received no specific funding for this work.*<br><br>**Funded studies**<br>Enter a statement with the following details:<br>• Initials of the authors who received each award<br>• Grant numbers awarded to each author<br>• The full name of each funder<br>• URL of each funder website<br>• Did the sponsors or funders play any role in the study design, data collection and analysis, decision to publish, or preparation of the manuscript?<br>• **NO** - Include this sentence at the end of your statement: *The funders had no role in study design, data collection and analysis, decision to publish, or preparation of the manuscript.*<br>• **YES** - Specify the role(s) played.<br><br>* typeset | |
| **Competing Interests**<br><br>Use the instructions below to enter a competing interest statement for this submission. On behalf of all authors, disclose any competing interests that could be perceived to bias this work—acknowledging all financial support and any other relevant financial or non-financial competing interests.<br><br>This statement is required for submission and **will appear in the published article** if the submission is accepted. Please make sure it is accurate and that any funding sources listed in your Funding Information later in the submission form are also | The authors have declared that no competing interests exist. |

| | |
|---|---|
| declared in your Financial Disclosure statement.<br><br>View published research articles from *PLOS ONE* for specific examples.<br><br>**NO authors have competing interests**<br><br>Enter: *The authors have declared that no competing interests exist.*<br><br>**Authors with competing interests**<br><br>Enter competing interest details beginning with this statement:<br><br>*I have read the journal's policy and the authors of this manuscript have the following competing interests: [insert competing interests here]*<br><br>* typeset | |
| **Ethics Statement**<br><br>Enter an ethics statement for this submission. This statement is required if the study involved:<br><br>• Human participants<br>• Human specimens or tissue<br>• Vertebrate animals or cephalopods<br>• Vertebrate embryos or tissues<br>• Field research<br><br>Write "N/A" if the submission does not require an ethics statement.<br><br>General guidance is provided below. Consult the submission guidelines for detailed instructions. **Make sure that all information entered here is included in the Methods section of the manuscript.** | Ethical approval for this study was approved by the Committee on Human Research, Publications and Ethics, School of Medicine and Dentistry, Kwame Nkrumah University of Science and Technology with a reference number CHRPE/AP/579/22. Approval was sought from the management of the Tatale district hospital |

| | |
|---|---|
| **Format for specific study types**<br><br>**Human Subject Research (involving human participants and/or tissue)**<br>• Give the name of the institutional review board or ethics committee that approved the study<br>• Include the approval number and/or a statement indicating approval of this research<br>• Indicate the form of consent obtained (written/oral) or the reason that consent was not obtained (e.g. the data were analyzed anonymously)<br><br>**Animal Research (involving vertebrate animals, embryos or tissues)**<br>• Provide the name of the Institutional Animal Care and Use Committee (IACUC) or other relevant ethics board that reviewed the study protocol, and indicate whether they approved this research or granted a formal waiver of ethical approval<br>• Include an approval number if one was obtained<br>• If the study involved *non-human primates*, add *additional details* about animal welfare and steps taken to ameliorate suffering<br>• If anesthesia, euthanasia, or any kind of animal sacrifice is part of the study, include briefly which substances and/or methods were applied<br><br>**Field Research**<br><br>Include the following details if this study involves the collection of plant, animal, or other materials from a natural setting:<br>• Field permit number<br>• Name of the institution or relevant body that granted permission | |
| **Data Availability**<br><br>Authors are required to make all data underlying the findings described fully available, without restriction, and from the time of publication. PLOS allows rare exceptions to address legal and ethical concerns. See the PLOS Data Policy and FAQ for detailed information. | Yes - all data are fully available without restriction |

Powered by Editorial Manager® and ProduXion Manager® from Aries Systems Corporation

| | |
|---|---|
| A Data Availability Statement describing where the data can be found is required at submission. Your answers to this question constitute the Data Availability Statement and **will be published in the article**, if accepted.<br><br>**Important:** Stating 'data available on request from the author' is not sufficient. If your data are only available upon request, select 'No' for the first question and explain your exceptional situation in the text box.<br><br>Do the authors confirm that all data underlying the findings described in their manuscript are fully available without restriction? | |
| **Describe where the data may be found in full sentences. If you are copying our sample text, replace any instances of XXX with the appropriate details.**<br><br>• If the data are **held or will be held in a public repository**, include URLs, accession numbers or DOIs. If this information will only be available after acceptance, indicate this by ticking the box below. For example: *All XXX files are available from the XXX database (accession number(s) XXX, XXX.*).<br>• If the data are all contained **within the manuscript and/or Supporting Information files**, enter the following: *All relevant data are within the manuscript and its Supporting Information files.*<br>• If neither of these applies but you are able to provide **details of access elsewhere**, with or without limitations, please do so. For example:<br><br>*Data cannot be shared publicly because of [XXX]. Data are available from the XXX Institutional Data Access / Ethics Committee (contact via XXX) for researchers who meet the criteria for access to confidential data.*<br><br>*The data underlying the results presented in the study are available from (include the name of the third party* | All relevant data are within the manuscript and its Supporting Information files. |

| | |
|---|---|
| *and contact information or URL).*<br>• This text is appropriate if the data are owned by a third party and authors do not have permission to share the data.<br><br>* typeset | |
| Additional data availability information: | |

Powered by Editorial Manager® and ProduXion Manager® from Aries Systems Corporation

MANUSCRIPT Click here to access/download;Manuscript;Final_Submitted to Journal _MANUSCRIPT.docx

# Foetal outcomes and their correlates following caesarian section in a rural setting in Ghana

**Authors:**

Eugene Sackeya[1,2], Martin Muonibe Beru[1,3] Richard Nomo Angmortey[1,3], Douglas Aninng Opoku[4,5], Victoria Achiaa Boamah[6], Francis Appiah[4,7], Aliyu Mohammed[1]

Affiliations

1. Department of Epidemiology and Biostatistics, School of Public Health, Kwame Nkrumah University of Science and Technology, Kumasi-Ghana
2. Tatale District Hospital, Tatale-Ghana
3. Tamale Teaching Hospital, Tamale-Ghana
4. Department of Global Health and International Health, School of Public Health, Kwame Nkrumah University of Science and Technology, Ghana
5. Allen Clinic, Family Healthcare Services, Kumasi, Ghana
6. School of Health and Social Care, Swansea University,
7. Department of Social Sciences, Berekum College of Education, Berekum, Bono Region, Ghana

All correspondence should be addressed to Eugene Sackeya; mcsackeya@gmail.com

**Abstract**

**Background**: Regular evaluation of caesarean section (CS) is required due to the rising trend in CS and their outcomes. Many women recently opt for elective CS, even in resource-constraint settings. Data evaluating the outcomes of CS is however sparse. Hence, this study sought to determine the rate of perinatal foetal outcomes and their determinants following CS in the Tatale District Hospital of the Northern Region, Ghana.

**Methods**: A retrospective cross-sectional study was employed to analyze medical records of 275 women who underwent CS from 2019 to 2021. Data was collected from the hospital's record on CS cases from 2019 to 2021. Descriptive statistics were used to summarise the data and Pearson's chi-square/Fisher's exact test was used to examine the relationship between maternal and obstetric characteristics and foetal outcomes. At 95% confidence interval (95% CI), logistic regression was fitted to assess significant variables and reported the results using odds ratio.

**Results:** Of 1667 deliveries, 16.5% of the mothers gave birth by CS. Of this, 7.64% of babies were stillbirths. Babies born with poor Appearance, Pulse, Grimace, Activity and Respiration (APGAR) scores had an increased risk of infant mortality (AOR:523.19 95% Cl: 49.24-555.37). Having a history of previous CS, cephalopelvic disproportion and delayed labour were the major indictors for CS.

**Conclusion:** Overall, this study found a high rate of CS based on World Health Organization 's recommended CS rate. Interventions such as reducing the waiting time for surgery and early diagnosis of the need for CS, and ensuring the availability of modern equipment to resuscitate infants with low APGAR scores can significantly improve foetal outcomes following CS.

**Keywords:** Foetal outcomes; correlates; Caesarian section; Ghana; mortality

## Introduction

A life-saving surgical operation known as a caesarean section (CS) is performed when specific difficulties during pregnancy and labour occur [1]. It is a major procedure that carries acute maternal and perinatal dangers as well as potential long-term side effects that are still under investigation [1]. CS has increased dramatically worldwide in recent decades, especially in middle- and high-income countries [2]. The causes of this growth are complex and poorly understood. Economic, organizational, social, and cultural reasons, as well as changes in mother traits and professional practice approaches, have all been linked to this tendency [3]. Inequalities in the use of surgery, both between and within nations, as well as the expenditures that unneeded CS imposes on financially strapped health systems, are further issues and debates surrounding CS [4]. Worldwide, CS rates have been on the ascendancy from an estimated 7% in 1990 to 21% in 2021, and are expected to rise further in the coming decade [5]. CS rates in Eastern Asia (63%), Latin America and the Caribbean (54%), Western Asia (50%), Northern Africa (48%), Southern Europe (47%), and Australia and New Zealand (45%) have been predicted to have the highest rates by 2030 [6].

In resource-constrained areas such as sub-Saharan Africa, having access to safe CS is a key strategy for lowering maternal mortality [7]. Africa has the lowest CS rate in the world with only 7.3 % of newborns delivered through this method [8]. Egypt (51.8 %) and Mauritius (47 %) have the highest CS rates in Africa [8].

CS can effectively prevent maternal and perinatal death and morbidity when medically necessary [5]. However, there is little proof that caesarean birth is beneficial to women or infants who do not require it [6]. CS, like any surgery, can carry immediate and long-term risks that might last for years after delivery. Women who have inadequate access to comprehensive obstetric care are at higher risk [6]. It has been reported that women who are suspected of being in preterm labour, a planned CS (i.e., elective CS) delivery may be beneficial to the infant, but it can also be stressful for both mother and baby [9]. As a result, the best way to deliver preterm babies in both cephalic and breech positions is still up for debate [9,10]. For instance, there is evidence to the effect that CS delivery has been linked to neonatal fatalities and therefore, further research on the relationship between CS delivery and poor infant outcomes is needed [11]. Caesarean section has also been

linked to negative newborn outcomes such as gastrointestinal and respiratory problems, as well as mortality [12].

A review of national data from 159 countries indicated that countries with national CS delivery rates of 5% to 10% had the lowest neonatal and maternal mortality rates [1,13]. However, the benefits of CS delivery reduces when the rates exceed 10% [13]. This indicates that high CS delivery rates might not have additional benefits in terms of reducing mortality. In all regions, the WHO recommends that the caesarean delivery rate not exceed 15% of all deliveries [5]. Higher CS delivery rates was not associated with maternal or newborn mortality at a country level, according to a study across the 194 WHO member states [14]. Similar findings emerged from a sensitivity analysis that only included 76 nations with the best data on CS delivery rates: CS delivery rates more than 6.9 to 20.1 per 100 live births were negatively connected with the maternal death rate. Neonatal death was negatively linked with CS  rates, which ranged from 12.6 to 24.0 per 100 live births [14].

Only 4% of live births in Ghana are CS deliveries [15]. According to a survey by the Ghana Statistical Service (GSS), 11% of women who gave birth to a live baby in the two years before the survey delivered through CS. Regional variations in CS was observed with the Greater Accra region recording the highest rate, with nearly one in four women (23%) undergoing a CS. Volt[illegible] (15%), Ashanti (12%), Eastern (12%), Brong-Ahafo (12%), and Central (11%) are the other regions [15]. Between 1998 and 2014, Ghana had differences in the number of CS births, which grew over time [16]. A study also showed that fetal weight of 4kg and above along with referred pregnant women are the leading indications for CS in northern Ghana [17]. The majority of Ghanaian women (about 87%) choose vaginal birth over a CS p[illegible]edure. About 55% of those who had CS for their most recent birth had an elective rather than an emergency procedure [18]. In a study of women giving birth at the University of Cape Coast Hospital in Ghana, it was discovered that babies born via emergency CS had a considerably greater rate of adverse foetal outcomes [19]. Considering the complex situations of unmet needs and overuse of CS, coupled with unsafe surgical practices and the non-availability of essential medical equipment for CS operations [1], this study will provide insight into prevalence of neonatal fetal outcomes and their determinants. This could be useful for designing public health interventions targeted at CS births and neonatal and maternal health in Ghana.

## Methods

### Study design and setting

This was a retrospective analytical cross-sectional study that included 275 women who delivered through CS at the Tatale District Hospital, Ghana. The facility provides medical service to the residents of Tatale Sanguli district, parts of the Republic of Togo, and other neighbouring districts in the northern region of Ghana. It also serves as first point of call for all emergency cases from all sub-district facilities. Due to inadequate number of trained midwives in the district, community health nurses augment the efforts of the midwives. The hospital has consistently had only one medical officer who attends to surgical cases such as CS.

The Tatale District Hospital is limited in terms of advanced medical equipment. The facility lacks ultrasonographic machines for diagnosing and assessing pregnancy states in addition to non-availability of a Neonatal Intensive Care Unit (NICU), a monitoring machine for anaesthesia care and resuscitation devices and an adequate supply of oxygen. This can affect the quality CS conducted in the facility.

### Variables description

### Dependent variable

The key outcome variable was foetal outcomes. This was defined as children delivered either alive or dead.

### Independent variables

Ten (10) explanatory variables were selected for the study. These were APGAR, gravidity, parity and gestational age. APGAR score was a composite index obtained from five cardinal measures and were the appearance, pulse, grimace, activity, and respiration for first and fifth minutes after delivery. Gravidity indicates the number of times a participant has been pregnant prior to the current pregnancy whilst parity explains the number of times a participant has given birth prior to the current delivery**.** The gestational age describes the number of weeks a participant carried a pregnancy before delivery. Mother’s haemoglobin (HB) level indicates the HB level of the mother as recorded in the ANC book prior to labour and delivery, baby weight refers to the initial weight

recorded at birth whiles time between admission and CS refers to the period between the arrival of participants at the health facility and when the CS was done. Also, ANC visits indicate the number of times a mother attended antenatal clinic before delivery. Type of anaesthesia is defined here as whether a pregnant woman was given general anaesthesia or spinal anaesthsia prior to CS whiles type of CS indicates whether the surgery done was a planned one (elective) or emergency. These variables were selected due to their theoretical and practical significance to neonatal/foetal health [20,21] .

## Data Collection

The medical records of all caesarian section cases documented in the theatre register from 1st January, 2019 to 31st December, 2021 were reviewed. The perinatal fetal outcomes including the fetus/neonate coming out of surgery alive or dead following the caesarian section of all cases were retrieved from the theatre register. The key variables of interest extracted for the study included maternal age, parity, gravida, educational level, hemoglobin level and employment status. The type of CS delivery (emergency or elective CS), type of anesthesia (general or spinal), the fetal weight at birth and the time between admission and the CS were also retrieved from the theatre register. The data was retrieved using a checklist.

Data that were retrieved from the theatre register were compared with the labor room register and the antenatal register to ensure accuracy and also to track and fill all missing data from the records in the theatre.

## Statistical Analysis

STATA statistical software version 16.0 was used to analyze the data. Descriptive statistics using mean, standard deviation, frequencies, and percentages were used to describe categorical and continuous variables. Cros-tabulation computation was done for the outcome variable across the key explanatory variables. At a cut-off point of 0.05, a chi-square test of independence or Fisher exact test was calculated between the outcome variable and key explanatory variables and those that were not significant were not entered in the multivariate analysis. At 95% confidence interval (95% CI), two logistic regression models were fitted between the outcome variable and key

explanatory variables. The first model (Model I) explored a bivariate association whilst Model II followed a multivariate approach. The results were reported in crude odds ratio (COR) and adjusted odds ratio (AOR) for Model I and Model II respectively. An odd lesser than 1 was interpreted as having lesser likelihood of perinatal mortality whilst an odd above 1 was interpreted as having higher likelihood of perinatal mortality. A multi-collinearity test was performed for the key explanatory variables using the Variance Inflation Factor (VIF). The VIF results indicated no evidence of multi-collinearity between the explanatory variables (Mean VIF= 1.06, Maximum VIF = 1.12 Minimum VIF=1.02) (see Appendix 1). Finally, the Hosmer-Lemeshow test was applied to measure the model fit which indicated no evidence of poor fit (P=0.72).

## Ethical consideration

Ethical approval for this study was approved by the Committee on Human Research, Publications and Ethics, School of Medicine and Dentistry, Kwame Nkrumah University of Science and Technology with a reference number CHRPE/AP/579/22. Approval was sought from the management of the Tatale district hospital.

## Results

Study participants' characteristics and feotal outcome Table 1 reports on the study participants' characteristics and feotal outcome. A total of 1667 deliveries were recorded at the hospital within the period. The mean age of the mothers was 27.9 (±**7**.2). Out of the 1667 deliveries recorded, 275 (16.50%) were born by CS out of which 7.6% perinatal deaths were recorded. Majority (73.85%) of children delivered had APGAR score recordings from 8 to 10 within the first 5 minutes and about one-tenth (10.9%) recorded 0 to 3 APGAR score within the first 5 minutes after delivery. About one-fifth (19.3%) had their CS as a result of cephalopelvic disproportion (CPD).

From the bivariate analyses, baby's APGAR score was found to be associated with perinatal mortality. Other variables that were found to be associated with perinatal mortality included number of ANC visits by mothers, time between admission and CS as well as the baby's birth weight.

Table 1: Study participants' characteristics and Feotal outcome (N=275)

| Variable | Univariate | Bivariate | | | |
|---|---|---|---|---|---|
| | | Feotal outcome | | | |
| | **n (%)** | **Alive n (%)** | **Dead n (%)** | **$X^2$** | **(p-value)** |
| **Age Group** | | | | | **0.876*** |
| 15-19 | 28 (10.2) | 27 (96.4) | 1 (3.6) | | |
| 20-34 | 187 (68.0) | 172 (92.0) | 15 (8.0) | | |
| 35-49 | 83 (21.8) | 55 (91.7) | 5 (8.3) | | |
| Mean age (±SD) | **27.9 (±7.2)** | | | | |
| **NHIS Status** | | | | | **0.382*** |
| Insured with NHIS | 269 (97.8) | 249 (92.6) | 20 (7.4) | | |
| Not insured with NHIS | 6 (2.2) | 5 (83.3) | 1 (16.7) | | |
| **Employment status** | | | | **0.12** | **0.726** |
| Employed | 187 (68) | 172 (92.0) | 15 (8.0) | | |
| Unemployed | 88 (32) | 82 (93.2) | 6 (6.8) | | |
| **Occupation (n=187)** | | | | | **0.781*** |
| Civil servants | 4 (2.1) | 4 (100) | 0 (0.0) | | |
| Farmers | 110 (58.8) | 99 (90.0) | 11 (10.0) | | |
| Traders | 41 (21.9) | 39 (95.1) | 2 (4.9) | | |
| Skilled workers | 32 (17.1) | 30 (93.8) | 2 (6.2) | | |
| **Level of Education** | | | | | **0.941*** |
| No formal education | 185 (67.3) | 169 (91.4) | 16 (8.7) | | |
| Primary level | 14 (5.1) | 14 (100.0) | 0 (0.0) | | |
| JHS level | 29 (10.6) | 27 (93.1) | 2 (6.9) | | |
| SHS level | 38 (13.8) | 35 (92.1) | 3 (7.9) | | |
| Tertiary level | 9 (3.3) | 9 (100.0) | 0 (0.0) | | |
| **Gravidity** | | | | **0.14** | **0.708** |
| Primigravida | 74 (27.3) | 69 (93.2) | 5 (6.8) | | |
| Multigravida | 197 (72.7) | 181 (91.9) | 16 (8.1) | | |
| **Parity** | | | | | **0.798*** |
| Primiparous | 131 (48.3) | 122 (93.1) | 9 (6.9) | | |
| Multiparous | 84 (31.0) | 76 (90.9) | 8 (7.1) | | |
| Grand multiparous | 56 (20.7) | 52 (92.9) | 4 (7.1) | | |
| **Type of CS** | | | | | **0.618*** |
| Emergency CS | 269 (97.8) | 248 (92.2) | 21 (7.8) | | |
| Elective CS | 6 (2.2) | 6 (100.0) | 0 (0) | | |
| **Type of anesthesia** | | | | | **0.085*** |
| Spinal anesthesia | 252 (91.6) | 235 (93.3) | 17 (6.7) | | |
| General anesthesia | 23 (8.4) | 19 (82.6) | 4 (17.4) | | |
| **Baby APGAR score** | | | | | **0.000*** |
| 0-3 | 30 (10.9) | 10 (33.3) | 20 (66.7) | | |
| 4-10 | 245 (89.1) | 244 (99.6) | 1 (0.4) | | |
| **ANC visits** | | | | **7.27** | **0.007** |

| | | | | | |
|---|---|---|---|---|---|
| Less than 4 visit | 38 (13.8) | 31 (81.6) | 7 (18.4) | | |
| 4 or more visits | 237 (86.2) | 223 (94.1) | 14 (5.9) | | |
| **Gestational age** | | | | **4.75** | **0.029** |
| Pre-term | 75 (27.3) | 65 (86.7) | 10 (13.3) | | |
| Term/ Post term | 200 (72.7) | 189 (94.5) | 11 (5.5) | | |
| **Mother's HB level** | | | | | **0.031*** |
| Below 7.6 | 17 (6.2) | 13 (76.5) | 4 (23.5) | | |
| 7.6 and above | 258 (93.8) | 241 (93.4) | 17 (6.6) | | |
| **Time b/n admission and CS** | | | | | **0.002*** |
| Within 30 minutes | 11 (4.0) | 9 (81.8) | 2 (18.2) | | |
| Within 2 hours | 69 (25.1) | 58 (84.1) | 11 (15.9) | | |
| More than 2 hours | 195 (70.9) | 187 (95.9) | 8 (4.1) | | |
| **Baby's weight at birth** | | | | **6.65** | **0.031** |
| Low weight | 55 (20.0) | 47 (85.5) | 8 (14.5) | | |
| Normal weight | 220 (80.0) | 207 (94.1) | 13 (5.9) | | |

SD= standard deviation, NHIS= national health insurance scheme, JHS= junior high school, SHS= senior high school, CS=caesarean section, ANC= antenatal care, b/n= between, *= Analyzed using Fisher's exact test

**Indicators for CS**

Of the total number of CS, 19.3% were as a result of cephalopelvic disproportion. Also, 17.5% were due to mother's history of previous CS and delayed labour account for 15.3% of CS operations (Figure 1).

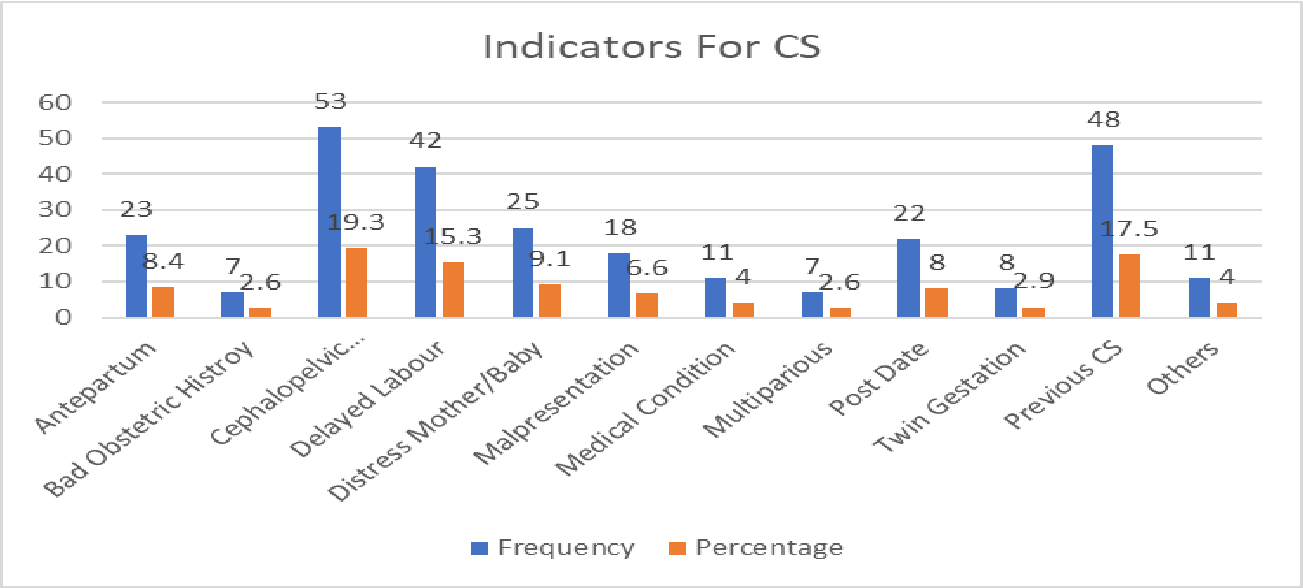


Figure 1: Indicators for Caesarean Section

## Foetal outcome by CS indicator

Figure 2 pictorially indicates foetal outcomes by their CS indicators. Almost 1 in every 10 perinatal mortalities (8.27%) occurred among mothers presented with antepartum haemorrahage. Meanwhile, postdated pregnancies, mothers with bad obstetric history and multiparity recorded no perinatal mortalities.

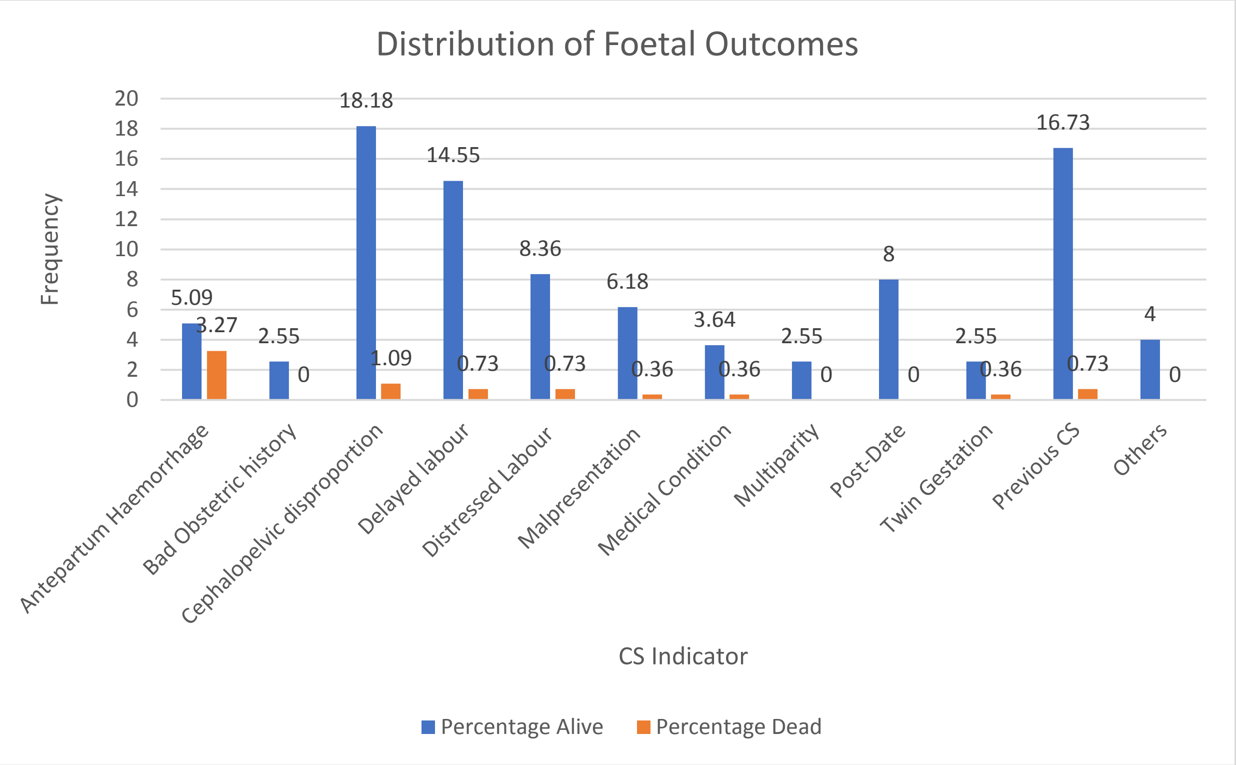


Figure 2: Distribution of fetal outcome by CS indicator

## Predictors Of Foetal Outcomes

Table 2 presents the results of the logistic regression of predictors of fetal outcomes. Children who were born preterm, mothers who visited the ANC less than 4 times, participants with HB level of 7.5g/dl, and women who had to wait more than two hours before the operation were found to be statistically associated with foetal deaths at the bivariate level (Model I). After adjusting for other covariates (Model II), children born with low APGAR score had higher odds of perinatal mortality compared to those with high/moderate APGAR score (AOR=523.19, 95%CI=49.24-555.37).

**Table 2: Logistic Regression results of predictors of foetal survival or death**

| | Model I | | Model II | |
|---|---|---|---|---|
| **Variables** | **COR** | **95%C.I (P-Value)** | **AOR** | **95%C.I(P-Value)** |
| **Baby APGAR score** | | | | |
| Moderate/high (4-10) | Ref | | | |
| Low (0-3) | 487.99 | [59.43-4007.32](0.000) | 523.19 | [49.24-555.37](0.000) |
| **ANC visits** | | | | |
| 4 or more visits | Ref | | | |
| Less than 4 visit | 3.60 | [1.35-9.60](0.011) | 2.68 | [0.39-18.65](0.319) |
| **Gestational age** | | | | |
| Term/Post term | Ref | | | |
| Pre-term | 2.64 | [1.07-6.51](0.035) | 1.61 | [0.29-8.95](0.586) |
| **Mother's HB(g/dl)** | | | | |
| 7.6 and above | Ref | | | |
| 7.5 and below | 4.36 | [1.28-14.83](0.018) | 6.28 | [0.30-8.94](0.236) |
| **Time b/n admission and CS** | | | | |
| More than 2 hours | Ref | | | |
| Within 2 hours | 4.43 | [1.70-11.55](0.002) | 0.95 | [0.19-4.86](0.951) |
| Within 30 minutes | 5.19 | [0.96-28.07](0.056) | 0.88 | [0.05-15.90](0.929) |
| **Baby weight at birth(kg)** | | | | |
| **Normal weight** | Ref | | | |
| Low weight | 2.71 | [1.06-6.91](0.037) | 0.89 | [0.14-5.56](0.901) |
| Number of observations | | **275** | | |
| Model Fit Testing (Hosmer-Lemeshow) | | **2.87(0.72)** | | |

95% confidence intervals in brackets, COR=Crude Odd Ratio, AOR = Adjusted Odds Ratio, Ref=Reference category

## DISCUSSION

The remarkable and consistent growth in the prevalence of CS over the past few decades has prompted more inquiry, discussion, and worry among healthcare professionals, governments, legislators, scientists, and clinicians [1,22].

The rate of CS (16.5%) in the present study is high compared with the 10% to 15.0% recommended by the WHO [23] but similar to the national rate (16.0%) that was reported in the Ghana Demographic Health Survey and an earlier study (16.0%) in 2017 [24]. However, the CS rate observed in the present study is lower than the 26.9% reported in a study in the Cape Coast ,Ghana [19]. The high rates of CS above WHO recommendation in this study may be due to the hospital

serving as a referral center for clinics, health centers, and Community-based Health Planning and Services (CHPS) compounds, as well as referrals from neighboring districts.

There was 7.6% of perinatal mortalities following CS within the study period. This rate was found to be relatively higher compared to a study conducted in Ethiopia, which found 5.6% perinatal deaths [25]. The difference in the mortality rate can be attributed to the differences available in the two facilities. For instance, the Ethiopian study was conducted in a facility with Neonatal Intensive Care Unit (NICU) while the setting of the present study does not have it. The perinatal mortality rate in the Tatale District Hospital following CS was also higher when compared with rates among women with combined delivery methods in a study that sort to compare if facility birth reduced maternal and perinatal mortality in the Brong Ahafo, Ghana [26]. The reason for this variation could be that most of the facilities within the Brong Ahafo have more skilled staff and modern equipment compared to the Tatale District Hospital.

The most common indicators for CS in the district hospital included cephalopelvic disproportion, previous CS, delayed labor and distressed mother/baby. These finding are relatively similar to earlier studies in Ghana that had previous CS, big baby, failure in progress and fetal distress being the most common indicators for CS [19]. Similar to a study conducted in a teaching hospital in Burkina Faso our study found that women who suffered antepartum hemorrhage recorded the highest perinatal mortalities making it the most dangerous indicator requiring early diagnosis and intervention [27] hence the need to train midwives, nurses and other practitioners on early sign of antepartum hemorrhage and its management.

The study revealed that children born with APGAR score of 0 to 3 within the first 5 minutes had a higher odd of prenatal mortality compared with children with 4 and above APGAR score. This finding is similar to a study that sort to find the association between APGAR scores of 7 to 9 and neonatal mortality and morbidity in a population based cohort study of term infants in Sweden [28]. According to the American society of pediatrics, "The Apgar score is an expression of the infant's physiologic condition at one point in time, which includes subjective components''. There are numerous factors that can influence the Apgar score, including maternal sedation or anesthesia, congenital malformations, gestational age, trauma, and interobserver variability. The incidence of low Apgar scores is inversely related to birth weight, and a low score cannot predict morbidity or

mortality for any individual infant. As previously stated, it also is inappropriate to use an Apgar score alone to diagnose asphyxia"[29].

**Strength and limitation of the study**

However, findings in the study are interpreted in the light of the following. Because this is a retrospective study, several critical obstetric outcomes and indications may not have been consistently reported, preventing further sub analysis. As an index study to determine the foetal outcomes following CS at the facility level, the findings give considerable evidence for the current situation and serve as the foundation for future research.

This study was conducted in one setting and thus findings cannot be generalized to represent other health facilities in Ghana, and another notable is the relatively small sample size, which reduces the statistical power and the presence of missing data. One other limitation is the large confidence intervals. This means the results with large confidence intervals should be interpreted with caution as it reduces precision.

**Conclusion**

The present study recorded high rates of CS (16.5%) and perinatal mortality (7.6%) following CS. The study found APGAR score below 4 to be significantly associated with prenatal death**.** We recommend a refresher training on early signs and effective management for all midwives and other auxiliary staff who augment the labor work force as well as establishment of a sonography department equipped with the necessary equipment and personnel to assist in the early diagnoses of cases for prompt intervention.

We also recommend an establishment of a functional neonatal intensive care unit. Meanwhile a multi-sector collaboration is important to address some of the challenges i.e., ANC attendance, low birth weight, early reporting to hospital and issues relating to maternal hemoglobin levels.

**Acknowledgements**

We are very grateful to all mothers whose records were reviewed for this piece of work. To the staff of the maternity ward, the ANC unit and theater we thank them for their efforts in saving lives and the documentation without which this study would not have been possible. We are also very

grateful to the management of the hospital and the District Director of Health Services for their approval and reviews. Lastly, we are grateful to God for the inspiration, guidance and protection throughout the study.

## Author Contributions

**Conceptualization:** ES, MMB, RNA & DAO

**Data curation:** ES, MMB, RNA, VAB, FA, DAO & AM

**Formal analysis:** ES, MMB, & RNA

**Methodology:** ES, MMB, RNA, VAB, FA, DAO & AM

**Project administration:** ES, MMB, & RNA

**Supervision:** DAO & AM

**Validation:** ES, MMB, RNA, VAB, FA, DAO & AM

**Writing – original draft:** ES, MMB, RNA, VAB, FA, DAO & AM

**Writing – review and editing:** ES, MMB, RNA, VAB, FA, DAO & AM

## Competing interest

The authors declare that no competing interest existed for this study.

## Funding

None

## Reference

1. Betrán AP, Ye J, Moller AB, Zhang J, Gülmezoglu AM, Torloni MR. The Increasing Trend in Caesarean Section Rates: Global, Regional and National Estimates: 1990-2014. Zeeb H, editor. PLOS ONE. 2016 Feb 5;11(2):e0148343.

2. Betran AP, Ye J, Moller AB, Souza JP, Zhang J. Trends and projections of caesarean section rates: global and regional estimates. BMJ Glob Health. 2021 Jun 1;6(6):e005671.

3. Ramos-Casals M, Brito-Zerón P, López-Guillermo A, Khamashta M, Bosch X. Department of error. The Lancet. 2014 Apr 26;383(9927):1464.

4. Ross MG, Jessie M, Amaya K, Matushewski B, Durosier LD, Frasch MG, et al. Correlation of arterial fetal base deficit and lactate changes with severity of variable heart rate decelerations in the near-term ovine fetus. Am J Obstet Gynecol. 2013 Apr;208(4):285.e1-6.

5. World Health Organization. WHO Statement on Caesarean Section Rates [Internet]. 2015. Available from: https://apps.who.int/iris/bitstream/handle/10665/161442/WHO_RHR_15.02_eng.pdf [Accessed January 2023]

6. Limbs & Things. Increasing Rates of Caesarean Section: Implications for Training [Internet]. Limbs & Things. 2022. Available from: https://limbsandthings.com/global/news/36013/increasing-caesarean-section-rates-and-the-implications-for-training [Accessed January 2023]

7. Yaya S, Bishwajit G, Okonofua F, Uthman O. Under five mortality patterns and associated maternal risk factors in sub-Saharan Africa: A multi-country analysis. PLOS ONE. 2018 Oct 25;13:e0205977.

8. Masukume G, Maswime S. Increasing caesarean sections in Africa could save more mothers' lives. The Conversation. 2017

9. Alfirevic Z, Milan SJ, Livio S. Caesarean section versus vaginal delivery for preterm birth in singletons. Cochrane Database Syst Rev. 2012 Jun 13;(6):CD000078.

10. Toijonen A, Hinnenberg P, Gissler M, Heinonen S, Macharey G. Maternal and neonatal outcomes in the following delivery after previous preterm caesarean breech birth: a national cohort study. J Obstet Gynaecol. 2022 Jan 2;42(1):49–54.

11. Degu Ayele A, Getnet Kassa B, Nibret Mihretie G, Yenealem Beyene F. Decision to Delivery Interval, Fetal Outcomes and Its Factors Among Emergency Caesarean Section Deliveries at South Gondar Zone Hospitals, Northwest Ethiopia: Retrospective Cross-Sectional Study, 2020. Int J Womens Health. 2021;13:395–403.

12. Kyu HH, Shannon HS, Georgiades K, Boyle MH. Association of Urban Slum Residency with Infant Mortality and Child Stunting in Low and Middle Income Countries. BioMed Res Int. 2013 Sep 17;2013:e604974.

13. Diallo MH, Baldé IS, Diallo AD, Baldé O, Diallo BS, Sylla I, et al. Maternal Complications of Caesarean Section in a Resource-Limited Country: The Case of the Maternity Unit of Kankan Regional Hospital, Guinea. Open J Obstet Gynecol. 2019;09(07):981–90.

14. Molina G, Weiser TG, Lipsitz SR, Esquivel MM, Uribe-Leitz T, Azad T, et al. Relationship Between Cesarean Delivery Rate and Maternal and Neonatal Mortality. JAMA. 2015 Dec 1;314(21):2263–70.

15. Seidu AA, Hagan JE, Agbemavi W, Ahinkorah BO, Nartey EB, Budu E, et al. Not just numbers: beyond counting caesarean deliveries to understanding their determinants in Ghana using a population based cross-sectional study. BMC Pregnancy Childbirth. 2020 Feb 18;20(1):114.

16. Aboagye RG, Boah M, Okyere J, Ahinkorah BO, Seidu AA, Ameyaw EK, et al. Mother and newborn skin-to-skin contact in sub-Saharan Africa: prevalence and predictors. BMJ Glob Health. 2022 Mar 1;7(3):e007731.

17. Apanga PA, Awoonor-Williams JK. Predictors of caesarean section in Northern Ghana: a case-control study. Pan Afr Med J. 2018 Sep 1;29(20).

18. Banchani E, Tenkorang EY. Determinants of Low Birth Weight in Ghana: Does Quality of Antenatal Care Matter? Matern Child Health J. 2020 May 1;24(5):668–78.

19. Prah J, Kudom A, Afrifa A, Abdulai M, Sirikyi I, Abu E. Caesarean section in a primary health facility in Ghana: Clinical indications and feto-maternal outcomes. J Public Health Afr. 2017 Dec 1;8.

20. Madaj B, Smith H, Mathai M, Roos N, van den Broek N. Developing global indicators for quality of maternal and newborn care: a feasibility assessment. Bull World Health Organ. 2017 Jun 1;95(6):445-452I.

21. Saturno-Hernández PJ, Martínez-Nicolás I, Moreno-Zegbe E, Fernández-Elorriaga M, Poblano-Verástegui O. Indicators for monitoring maternal and neonatal quality care: a systematic review. BMC Pregnancy Childbirth. 2019 Jan 11;19(1):25.

22. Brüggmann D, Löhlein LK, Louwen F, Quarcoo D, Jaque J, Klingelhöfer D, et al. Caesarean Section--A Density-Equalizing Mapping Study to Depict Its Global Research Architecture. Int J Environ Res Public Health. 2015 Nov 17;12(11):14690–708.

23. Betrán AP, Ye J, Moller AB, Zhang J, Gülmezoglu AM, Torloni MR. The Increasing Trend in Caesarean Section Rates: Global, Regional and National Estimates: 1990-2014. PloS One. 2016;11(2):e0148343.

24. DHS. Ghana Special Demographic and Health Survey 2017 | GHDx [Internet]. 2017. Available from: https://ghdx.healthdata.org/record/ghana-special-demographic-and-health-survey-2017 [Accessed December 2022]

25. Elias S, Wolde Z, Tantu T, Gunta M, Zewudu D. Determinants of early neonatal outcomes after emergency cesarean delivery at Hawassa University comprehensive specialised hospital, Hawassa, Ethiopia. PLOS ONE. 2022 Mar 24;17(3):e0263837.

26. Gabrysch S, Nesbitt RC, Schoeps A, Hurt L, Soremekun S, Edmond K, et al. Does facility birth reduce maternal and perinatal mortality in Brong Ahafo, Ghana? A secondary analysis using data on 119 244 pregnancies from two cluster-randomised controlled trials. Lancet Glob Health. 2019 Aug 1;7(8):e1074–87.

27. Lankoande M, Bonkoungou P, Ouandaogo S, Dayamba M, Ouedraogo A, Veyckmans F, et al. Incidence and outcome of severe ante-partum hemorrhage at the Teaching Hospital Yalgado Ouédraogo in Burkina Faso. BMC Emerg Med. 2017 May 31;17(1):17.

28. Razaz N, Cnattingius S, Joseph KS. Association between Apgar scores of 7 to 9 and neonatal mortality and morbidity: population based cohort study of term infants in Sweden. BMJ. 2019 May 7;365:l1656.

29. American Academy of Pediatrics. The Apgar Score [Internet]. 2022. Available from: https://www.acog.org/en/clinical/clinical-guidance/committee-opinion/articles/2015/10/the-apgar-score [Accessed January 2023]

Appendix 1

Click here to access/download
**Supporting Information**
Appendix 1.docx

Dataset

Click here to access/download
**Supporting Information**
CS_DATASETS.xlsx
