# Supplementary material for: Fetal outcomes and their correlates following caesarian section in a rural setting in Ghana
Source: PLoS One. 2023 Oct 31;18(10):e0293029. doi: 10.1371/journal.pone.0293029 (PMC10617686; doi:10.1371/journal.pone.0293029)
Supplement: Attachment — Submitted filename: RESPONSES TO REVIEWERS COMMENTS.pdf [file pone.0293029.s004.pdf]

ALLEN CLINIC
FAMILY HEALTHCARE SERVICES
P. O. Box X543 FNT, Kumasi, Ghana
Tel: 0322493429 | 0507171650
Loc: Ahensan Estates near KNUST Gate, Kumasi
Email: allenfhs@gmail.com

RESPONSES TO REVIEWERS' COMMENTS – FETAL OUTCOMES AND THEIR CORRELATES FOLLOWING CAESARIAN SECTION IN A RURAL SETTING IN GHANA *(MANUSCRIPT ID.* PONE-D-23-05404*).*

17th July 2023

The Editor-In-Chief
PLOS One

Section of Paper	Reviewers' Comments	Response from Authors
**Academic Editor**		
General comments	Please provide additional details regarding participant consent. In the ethics statement in the Methods and online submission information, please ensure that you have specified (1) whether consent was informed and (2) what type you obtained (for instance, written or verbal, and if verbal, how it was documented and witnessed). If your study included minors, state whether you obtained consent from parents or guardians. If the need for consent was waived by the ethics committee, please include this information.	This has been done as suggested<br><br>*…Since this was a retrospective study using secondary data, informed consent was not obtained from the participants and this was made clear during the application for the ethical clearance.*
	If you are reporting a retrospective study of medical records or archived samples, please ensure that you have discussed whether all data were fully anonymized before you accessed them and/or whether the IRB or ethics committee waived the requirement for informed consent. If	Informed consent was not sought in this study because no physical contact with the study participants was made during the course of the data collection and this was made clear during the ethical clearance application process to the Ethics Committee. All the study data were

	patients provided informed written consent to have data from their medical records used in research, please include this information.	anonymized and this was also explained in the ethics application.<br><br>*...Since this was a retrospective study using secondary data, informed consent was not obtained from the participants and this was made clear during the application for the ethical clearance*.
**Reviewer 1**		
Introduction	This is a well written manuscript which describes the outcomes among 275 c sections at a District Hospital in Ghana.<br>In general it is well written and contributes some important insights by way of contributing a detailed look at c section outcomes in one facility.	Thank you
	The paper uses a variety of terms and should more precisely define the period of observation so that the results can be compared to national and international stillbirth and neonatal or perinatal mortality rates. I think these should be reported as rates per 1000 births for comparability.	The period of observation was stated in the data collection section as "The medical records of all caesarian section cases documented in the theatre register from 1st January 2019 to 31st December 2021 were reviewed". In most rural settings in Ghana, medical officer availability in rural facilities in a big challenge and so the authors considered this period because at the time there had been consistency in the availability of medical officers who have the skills to render the service of CS to clients who may require the service hence the need to evaluate the service during that period. We have also considered converting the mortality rates to per 1000 to make it comparable with national and international rates as recommended.

**Results**	The analysis generally seems sound, but in one instance the interpretation seems inconsistent with the data presented - this is in regards to mortality rates for women who waited more or less than 2 hours. The conclusions made seem logical but do not align with the data shown - possibly due to small sample size or some other underlying dynamic	This is true as our interpretation of women who waited for more than two hours to be associated with mortality is inconsistent with what was presented in the bivariate table and hence has been corrected. The demand for CS in the setting is mostly that of emergency, women report late to the health facility or are referred from lower facilities but due to transportation and other related problems, they arrive late and so even though surgery is seen to be within 30minutes of arrival, the condition is already bad compared to those who came to the hospital in time and were monitored for some time before the need for a surgical intervention was decided.<br><br>*…Women who were admitted within 30 minutes before the CS due to delays in reporting to the hospital in time were found to be statistically associated with fetal mortalities at the bivariate level.*
**Conclusion**	Similarly, the conclusions and recommendations seem like they don't necessarily flow from the data presented. I would recommend giving additional thought to what this data, which is limited by its nature as a retrospectively extracted dataset does show - namely that there were high rates of stillbirth and mortality following c-section - which ideally could be better separated? The data do seem to indicate that anemic mothers with less than 4 ANC carrying small and preterm infants are more likely to lose them, at least in this setting. The conclusion	We agree that some of the recommendations made did not flow from the finding including the need for a sonography department equipped with the necessary equipment and personnel to assist in the early diagnoses of cases for prompt intervention and the establishment of a functional neonatal intensive care unit and hence have been addressed.<br><br>*…The present study recorded high rates of CS (16.5%) and fetal mortality of 76.4 per 1000 total births following CS. The study found APGAR score below 4 to be significantly associated with fetal mortalities.* ***We*** *recommend a refresher training on early signs and*

	that a NICU is needed may follow, if the proportion of infants who are preterm and delivered by CS (~27%) is reflective of the overall prematurity rate, but that is unlikely and not established.	*effective management of labor for all midwives and other auxiliary staff who augment the labor work force.* *We also recommend a multi-sector collaboration to address some of the challenges identified i.e., ANC attendance, low birth weight, early reporting to hospital and issues relating to maternal hemoglobin levels.*
**Reviewer 2**		
**Abstract**	What is the definition of poor APGAR score? What is the cut-off?	What we meant was the category of APGAR score marked 0-3 in the fifth minute. To address this, the word poor has been replace.<br><br>*...An APGAR score of 4 and above was considered to be moderate to high score whiles a score of 0-3 was considered a poor score.*
	What does "perinatal fetal outcome" mean? Is it death, complications, or both?	This has been duly changed to fetal mortality. Our focus has been on the mortality and does not include any other complications.<br><br>*...Hence, this study sought to determine the rate of fetal mortalities and their determinants following CS in the Tatale District Hospital of the Northern Region, Ghana.*
	(AOR:523.19 95% Cl: 49.24-555.37). The stated adjusted OR with its CI is not right. It should be rechecked.	this has been rechecked, we acknowledge a missing value, in the table the upper limit confidence interval was misquoted i.e. 555.37 instead of 5559.37 and this has been corrected and the structure rearranged to alien with standard writing formats.

		...Babies born with fifth-minute Appearance, Pulse, Grimace, Activity and Respiration (APGAR) scores of (0-3) had an increased risk of fetal mortality (AOR = 523.19, 95%CI: 49.24–5559.37, p = <0.001).
**Introduction**	The introduction is wordy, and authors should consider cutting down to about a half to two-thirds.	This has been done as suggested
	There is no statement of specific objectives, including any pre-specified hypothesis.	This has been provided in the introduction as suggested. The objective of the study was…<br><br>*...To determine the prevalence and factors associated with fetal mortality following CS. This study also sought to estimate the prevalence of CS at the Tatale District Hospital.*
**Methods**	Line 136 “these were APGAR”, which minute APGAR was that? It should be specific as APGAR is done at specific times after birth.	This has been duly rectified as the fifth minute APGAR score was considered in the analysis.<br><br>*...These were APGAR score at fifth minute, gravidity, parity and gestational age.*
	Line 142-143: at what point was the HB picked? Which one was used if the prospective mother had multiple HBs checked?	in preparing the women for the surgical procedure, one of the requirement is to check the HB level and blood grouping, the reason being that there may be the need for transfusion during the surgery and so the last HB that was checked before the women are taken in for the surgical procedure was used and have been corrected as recommended.<br><br>*...Mother’s hemoglobin (HB) level indicates the HB level of the mother as recorded in the theater records book,*

		specifically the HB taken during the process of preparing the woman for the CS.
	What were the criteria for the inclusion of some specific records into the study?	In view of the retrospective nature, the possibility of missing data was anticipated and so variables with missing data ten percent or less were considered. In the write up, we acknowledge an omission and that has been corrected accordingly.<br><br>*...Only variables with missing values less than ten percent were considered with missing values manually imputed using either the mean, median or the mode where appropriate.*
	How was the sample size determined? Why did the authors choose those 3 years and not 2 or 4 years?	The sample size was determined using the census technique and the study period was purposefully selected due to the availability of medical officers to render the service CS throughout the years. The three years chosen is believed will give a fair estimate of the occurrence of the health event as the average of the three years is what will be calculated. This we have tried to spell out in the current write up.<br><br>*...The census technique was used to review medical records of all CS cases documented in the theatre register from 1st January, 2019 to 31st December, 2021. This period was purposively selected to assess the fetal mortalities following CS of all cases within the period. The reason for selecting this period was the constant availability of medical officers qualified to provide CS service to pregnant women who may need it.*

	There is nothing about data capture, data management and software used. These should be specified.	We acknowledge this and have duly capture that in the current write up.<br><br>*...The data were retrieved using a predesign checklist (designed using the Microsoft excel software).*<br><br>*...data was then saved in a computer hard-drive and also in a Google drive for use and protection.*
	What was the percentage of missing values and how were they dealt with in data analysis?	We strongly acknowledge this also and have duly capture that in the current write up.<br><br>*...Only variables with missing values less than ten percent were considered with missing values manually imputed using either the mean, median or the mode where appropriate.*
	Line 169: Indicate that 0.05 is the p-value.	This has been addressed as recommended.<br><br>*...At a cut-off p-value of ≤0.05.*
**Result**	Line 194, 195 & 197: Is this at 5 minutes or within the first 5 mins? APGAR is done typically at 1 min and 5 mins. The authors should be specific.	We acknowledge that the APGAR score is taken in the first and fifth minute after birth. Whiles information on both first and fifth minute APGAR scores are available, we used the fifth minute APGAR score for the purpose of this analysis and so the correction have been effected as recommended.<br><br>*...Majority (89.1%) of children delivered had APGAR score recordings from 4 to 10 in the fifth-minute and*

		about one-tenth (10.9%) recorded 0 to 3 APGAR score in the fifth-minute after delivery.
	All p-values written as 0.000 should be converted to <0.001. P-value cannot be 0.	This has been duly corrected as recommended
	Figures I and II should be converted to a table. The information would be simpler as such.	All two figures have been converted to tables us recommended.
	Line 288: The OR with CI combination is wrong. They should be corrected as well as the same in the table.	Duly corrected<br><br>*...After adjusting for other covariates (Model II), children born with fifth minute APGAR score (0-3) had higher odds of fetal mortality compared to those with fifth minute APGAR score of 4-10 (AOR = 523.19, 95%CI: 49.24–5559.37, p = <0.001).*

We hereby submit our revised manuscript for your consideration.

Sincerely,

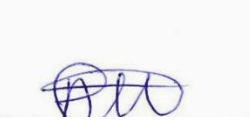

DOUGLAS ANINNG OPOKU
